# Grading Meningioma: A Comparative Study of Thallium-SPECT and FDG-PET

**DOI:** 10.1097/MD.0000000000000549

**Published:** 2015-02-13

**Authors:** Sachi Okuchi, Tomohisa Okada, Akira Yamamoto, Mitsunori Kanagaki, Yasutaka Fushimi, Tsutomu Okada, Moritaka Yamauchi, Masako Kataoka, Yoshiki Arakawa, Jun C. Takahashi, Sachiko Minamiguchi, Susumu Miyamoto, Kaori Togashi

**Affiliations:** From the Department of Diagnostic Imaging and Nuclear Medicine (SO, TO, AY, MK, YF, TO, MY, MK, KT); Department of Neurosurgery (YA, JCT, SM); and Department of Clinical Pathology (SM), Kyoto University Graduate School of Medicine, Kyoto, JAPAN.

## Abstract

The purpose was to compare capability of fluorine-18 fluorodeoxyglucose (FDG)-PET and thallium-201 (Tl)-SPECT for grading meningioma.

This retrospective study was conducted as a case-control study under approval by the institutional review board. In the hospital information system, 67 patients (22 men and 45 women) who had both FDG-PET and Tl-SPECT preoperative examinations were found with histopathologic diagnosis of meningioma. The maximum FDG uptake values of the tumors were measured, and they were standardized to the whole body (SUVmax) and normalized as gray matter ratio (SUVRmax). Mean and maximum Tl uptake ratios (TURmean and TURmax, respectively) of the tumors were measured and normalized as ratios to those of the contralateral normal brain. Receiver-operating characteristic curve analyses of the 4 indexes were conducted for differentiation between low- and high-grade meningiomas, and areas under the curves (AUCs) were compared. Correlation coefficients were calculated between these indexes and Ki-67.

Fifty-six meningiomas were classified as grade I (low grade), and 11 were grade II or III (high grade). In all 4 indexes, a significant difference was observed between low- and high-grade meningiomas (*P* < 0.05). AUCs were 0.817 (SUVmax), 0.781 (SUVRmax), 0.810 (TURmean), and 0.831 (TURmax), and no significant difference was observed among the indexes. Their sensitivity and specificity were 72.7% to 90.9% and 71.4% to 87.5%, respectively. Correlation of the 4 indexes to Ki-67 was statistically significant, but coefficients were relatively low (0.273–0.355).

Tl-SPECT, which can be used at hospitals without a cyclotron or an FDG distribution network, has high diagnostic capability of meningioma grades comparable to FDG-PET.

## INTRODUCTION

Meningioma is the most common benign intracranial tumor, accounting for about 13% to 26% of all primary intracranial tumors.^1^ World Health Organization (WHO) criteria classifies meningioma into grades I, II, and III,^2^ which had recurrence in 7%, 35%, and 73%, respectively.^3^ Some study reported that the elevated Ki-67 proliferation index has been associated with an increased recurrence rate.^4–7^ Once a meningioma recurs, it is more likely to recur again, resulting in a poor prognosis.^8–10^ Therefore, a noninvasive imaging method that can differentiate the histologic grades and evaluate biological aggressiveness, such as Ki-67, is desirable for presurgical planning of meningioma.

Previous studies evaluated intracranial meningioma with fluorine-18 fluorodeoxyglucose (FDG) positron emission tomography (PET), showing correlations between FDG uptake and histopathologic grades or biological aggressiveness of meningioma.^11–14^ A potential role of thallium-201 (Tl) single photon emission computed tomography (SPECT) has also been reported in patients with meningioma.^15–18^

FDG-PET examination is increasing, but its availability is still limited due to requirements of a cyclotron or a local FDG distribution network, as well as a relatively expensive PET system itself. On the other hand, Tl-SPECT examination is much widely available, although spatial resolution is lower than FDG-PET. So far as we could find, no comparative study of the 2 examinations has been conducted, and we do not know if one is better than the other or not. Therefore, the purpose of this study was to compare grading capability of the 2 examinations focusing on meningioma.

## MATERIALS AND METHODS

### Patients

This study was conducted as a case-control study under approval by the institutional review board. Informed consent was waived due to retrospective nature of this study. The hospital database was examined from October 2010 to April 2014 for patients who had both FDG-PET and Tl-SPECT examinations before biopsy or embolization, and was histopathologically diagnosed to have intracranial meningioma by surgical resection. Two patients who had meningioma with intraosseous or extracranial extension were not included.

A total of 67 patients (22 men and 45 women; range 33–86 years old, mean 60 years old) fulfilled the inclusion criteria. Among them, 61 patients were operated for the first time, and the remaining 6 patients had an operation for recurrence. Histopathologic grades of the meningioma were determined from grade I to grade III by the WHO classification 2007.^2^ They were sorted into low-grade (WHO grade I) or high-grade (WHO grades II and III) groups for further analysis.^14^ Furthermore, tumor proliferation index was evaluated by MIB-1 immunostaining, which is used for the assessment of Ki-67 expression in tumors. In 2 patients, MIB-1 immunostaining was not conducted, and Ki-67 index values were unavailable.

### Image Acquisition

The mean interval between scans and surgical resection of meningioma was 36 days (range 2–155 days) and 37 days (range 2–155 days) for FDG-PET and Tl-SPECT, respectively. The intervals between FDG-PET and Tl-SPECT were within 7 days.

FDG-PET scans were conducted using a PET/CT scanner (Discovery ST Elite; GE Healthcare, Waukesha, WI). Patients fasted for at least 4 hours prior to the scan. After intravenous administration of 4 MBq/kg of FDG, patients rested in a waiting room for 30 minutes. Emission scans of the brain were conducted for 15 minutes. Resolutions were 2.0 × 2.0 × 4.25 mm (47 slices).

Tl-SPECT was acquired at resting state using a 2-head rotating gamma camera (Infinia; GE Medical Systems, Milwaukee, WI) with an extended low-energy general-purpose collimator. After intravenous administration of 74 MBq of Tl-201, the scan was conducted 15 minutes later. Data were acquired through a 360° rotation at angle intervals of 6°, each for 20 seconds. Total imaging time was 20 minutes. Transverse reconstruction was conducted using ordered subset expectation maximization (subsets 10 and iterations 2), and resolutions were 4.42 × 4.42 × 4.42 mm (33–47 slices).

MR scans were conducted using 3T MR units (Magnetom Trio or Magnetom Skyra; Siemens, Erlangen, Germany) with a 32-channel head coil. Preoperative scans included 3-dimensional T1-weighted imaging in isotropic 0.7–0.9 mm resolution covering the whole brain before and after administration of a Gadolinium contrast agent (0.1 mmol/kg).

### Image Analysis

**FIGURE 1:**
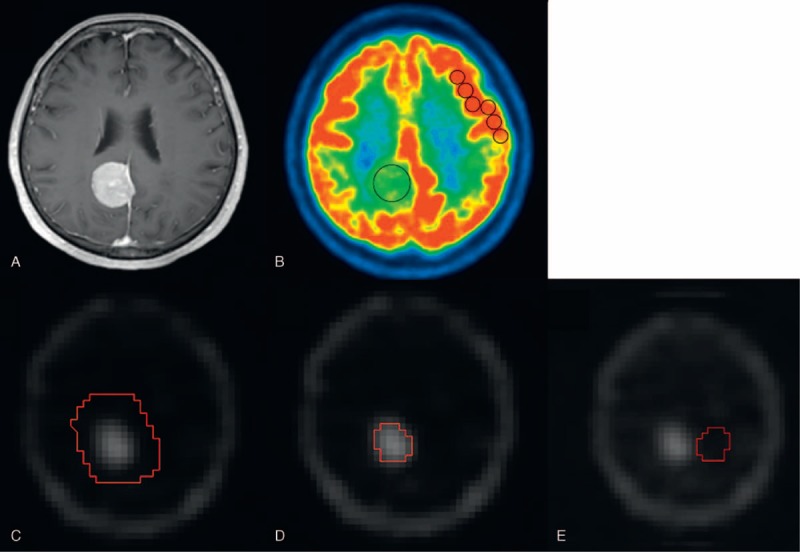
A representative analysis of a meningothelial meningioma (grade I), 70-year-old patient. Contrast-enhanced MRI (A) shows an enhanced extra-axial tumor at the right parietal region. FDG-PET (B) and Tl-SPECT (C–E) images show high uptake in the corresponding area. (B) On the FDG-PET image, ROIs were drawn on the tumor and contralateral gray matter. (C) On the Tl-SPECT image, the area corresponding to the tumor was encircled including the normal-appearing surroundings, but high uptake areas of the skull were excluded. (D) The tumor area way applying a threshold algorithm (Otsu method). (E) The tumor ROI was flipped horizontally, which was placed as the reference ROI at the contralateral normal brain of the same slice. By using these analysis procedures, SUVmax, SUVRmax, TURmean, and TURmax were measured as 5.75, 0.54, 6.84, and 9.30, respectively. FDG = fluorine-18 fluorodeoxyglucose, PET = positron emission tomography, ROI = region of interest, SPECT = single photon emission computed tomography, SUV = standardized uptake value, SUVmax = the maximum value of SUV, SUVRmax = the maximum SUV ratio, Tl = thallium-201, TURmax = the maximum Tl uptake ratio, TURmean = the mean Tl uptake ratio.

**FIGURE 2:**
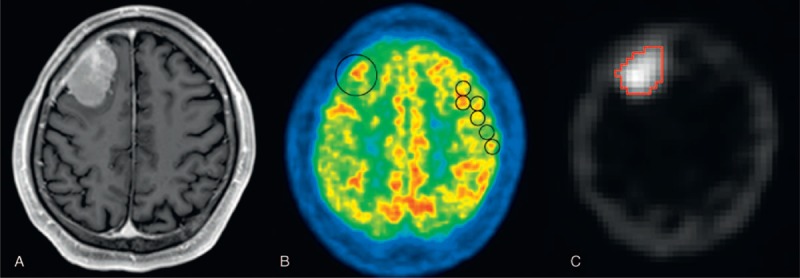
Contrast-enhanced MRI (A), FDG-PET (B), and Tl-SPECT (C) images of a 53-year-old patient with WHO grade II meningioma, atypical type. The FDG-PET image (B) and Tl-SPECT image (C) show high uptake in the tumor at the right frontal region. (B)The circles show examples of drawn ROI. (C)The tumor is selected with a threshold algorithm. The SUVmax, SUVRmax, TURmean, and TURmax were 8.69, 0.89, 11.92, and 16.57. FDG = fluorine-18 fluorodeoxyglucose, PET = positron emission tomography, ROI = region of interest, SPECT = single photon emission computed tomography, SUV = standardized uptake value, SUVmax = the maximum value of SUV, SUVRmax = the maximum SUV ratio, Tl = thallium-201, TURmax = the maximum Tl uptake ratio, TURmean = the mean Tl uptake ratio.

Maximum values of SUV (SUVmax) were measured by placing regions of interest (ROIs) of the tumor on FDG-PET images^14^ using a workstation (Advantage Windows; GE Healthcare, Waukesha, WI) (Figures 1B and 2B). When the tumor was indistinguishable from the brain parenchyma, ROIs were drawn with careful reference to the MR images (Figures 1A and 2A).^14^ SUVmax values of the tumors were normalized by those of the reference areas, and maximum SUV ratios (SUVRmax) were calculated. Five to 6 consecutive reference ROIs (10 mm diameter) were placed in the frontoparietal cortex contralateral to the tumor to measure maximum FDG uptake of the reference region (Figures 1B and 2B). When a tumor was located near the cerebellum, reference ROIs were placed in the ipsilateral frontoparietal cortex.^14^ When a tumor was found on the median line, they were placed at either side of the frontoparietal cortex that was considered less affected by the tumor.

On Tl-SPECT images, tumor ROIs were drawn on the slice where the tumor showed maximal diameters.^15^ Because tumor border could not be defined clearly, a tumor was encircled including the normal-appearing surroundings, but high Tl uptake areas of the skull were carefully excluded (Figure 1C). A threshold algorithm (Otsu method) was applied to select the tumor (Figures 1D and 2C)^19^ using ImageJ software ver. 1.48 (http://imagej.nih.gov/ij/), and mean and maximum Tl uptake values were measured. To normalize these values, a reference ROI was placed on the contralateral side of the tumor on the same slice by flipping the tumor ROI horizontally^15^ (Figure 1E). When we could not define a reference ROI on the same slice because the tumor was large or found in the median line, we defined it at a normal brain area on another slice. Mean and maximum values of a tumor were normalized by mean reference values, and the mean and maximum Tl uptake ratios (TURmean and TURmax, respectively) were calculated. Finally, tumor sizes were measured on contrast-enhanced MRI images using a workstation (Centricity PACS; GE Healthcare, Waukesha, WI). All the measurements were conducted independently by 2 radiologists (S.O. and M.Y. both with 7 years of experience).

### Statistical Analysis

Intraclass correlation coefficients (ICCs) were used to examine agreement between 2 evaluators. The measured values were averaged and used for further analysis. The Mann–Whitney test was used to examine differences between low and high grades for SUVmax, SUVRmax, TURmean, TURmax, and size. Age difference was compared between the 2 groups with 2-tailed 2-sample *t* test.

The receiver-operating characteristic (ROC) curve analyses were conducted for differentiation of low and high grades. Areas under the curves (AUCs) were calculated and compared among the 4 indexes with the method of DeLong et al.^20^ The optimal cutoff values were also determined by the values that maximized the Youden index (sensitivity + specificity − 1).^21^ To investigate relationship to Ki-67 proliferation index, Spearman correlation coefficients of the 4 indexes were also calculated and compared.

A *P* value less than 0.05 was considered statistically significant. Statistical analyses were conducted using a commercially available software (MedCalc ver. 13.3; MedCalc Software, Ostend, Belgium).

## RESULTS

Histopathologic diagnosis were grade I in 56 tumors (24 meningothelial, 17 fibrous, 10 transitional, 2 microcystic, 1 angiomatous, 1 meningothelial and angiomatous, and 1 psammomatous meningioma), grade II in 10 tumors (all atypical meningioma), and grade III in 1 tumor (anaplastic meningioma).

ICCs between 2 evaluators were 0.94 (95% CI 0.91–0.96), 0.90 (0.85–0.94), 0.83 (0.74–0.89), and 0.85 (0.77–0.90) for SUVmax, SUVRmax, TURmean, and TURmax, respectively. Agreements were almost perfect.^22^ In all 4 indexes, the values were significantly higher in high-grade meningioma than low-grade meningioma (*P* < 0.05 in all), and there was no statistically significant difference in age of the patients and tumor sizes between low and high grades (Table 1).

**TABLE 1:**
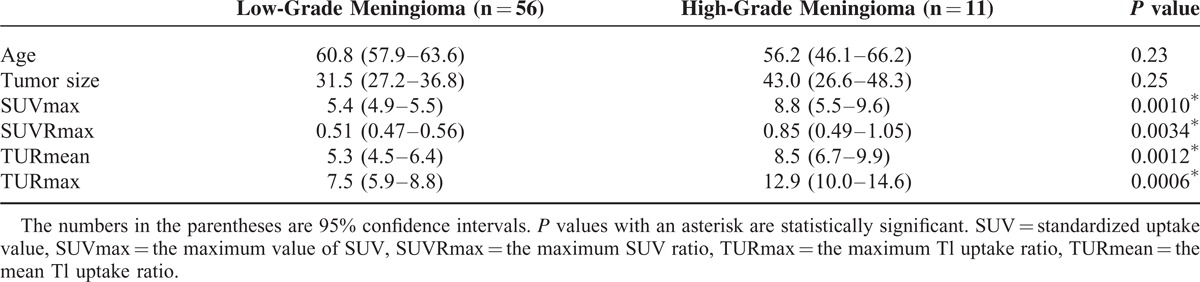
Comparisons of Low-Grade Meningioma (WHO Grade I) and High-Grade Meningioma (WHO Grade II and III)

**FIGURE 3:**
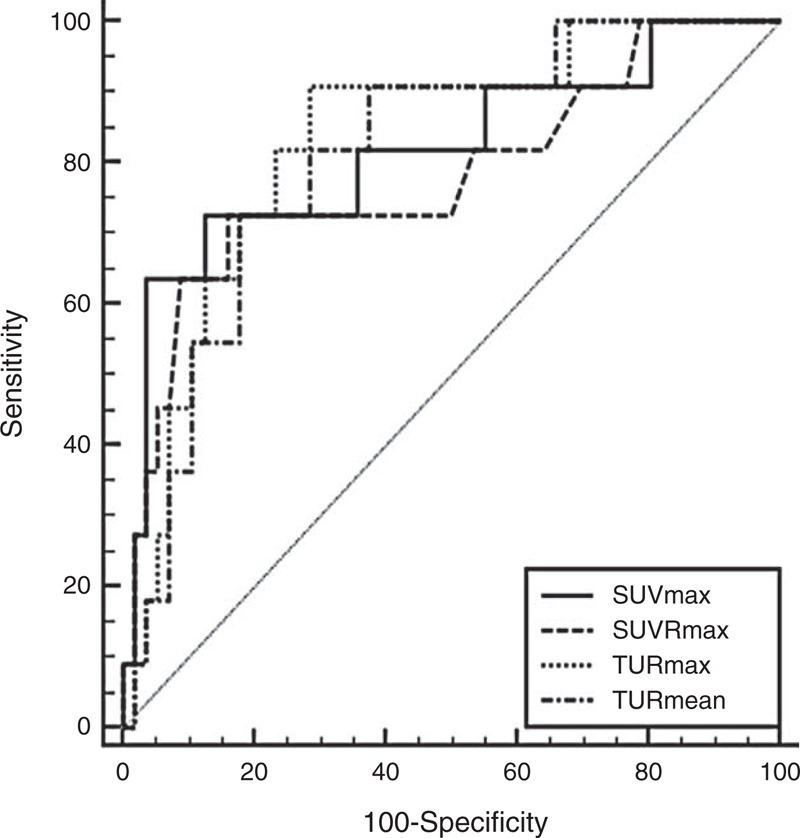
ROC curve analysis of SUVmax, SUVRmax, TURmean, and TURmax to differentiate low- and high-grade meningiomas. No statistically significant differences were observed among the AUCs. AUC = area under the curve, ROC = receiver-operating characteristic, SUV = standardized uptake value, SUVmax = the maximum value of SUV, SUVRmax = the maximum SUV ratio, TURmax = the maximum Tl uptake ratio, TURmean = the mean Tl uptake ratio.

In ROC curve analysis, AUCs were 0.817, 0.781, 0.810, and 0.831 for SUVmax, SUVRmax, TURmean, and TURmax, respectively. No statistically significant difference was observed among them (Figure 3). The sensitivity and specificity were 72.7% to 90.9% and 71.4% to 87.5%, respectively, when optimal cutoff values were applied (Table 2).

**TABLE 2:**
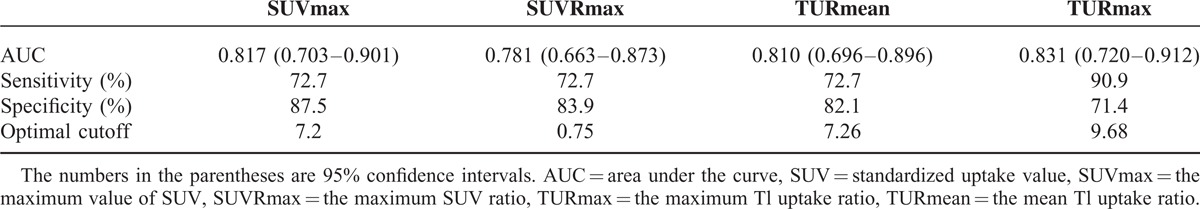
Receiver Operating Characteristic Curve Analysis for AUC, Sensitivity, Specificity and Optimal Cutoff Values of SUVmax, SUVRmax, TURmean, and TURmax

**FIGURE 4:**
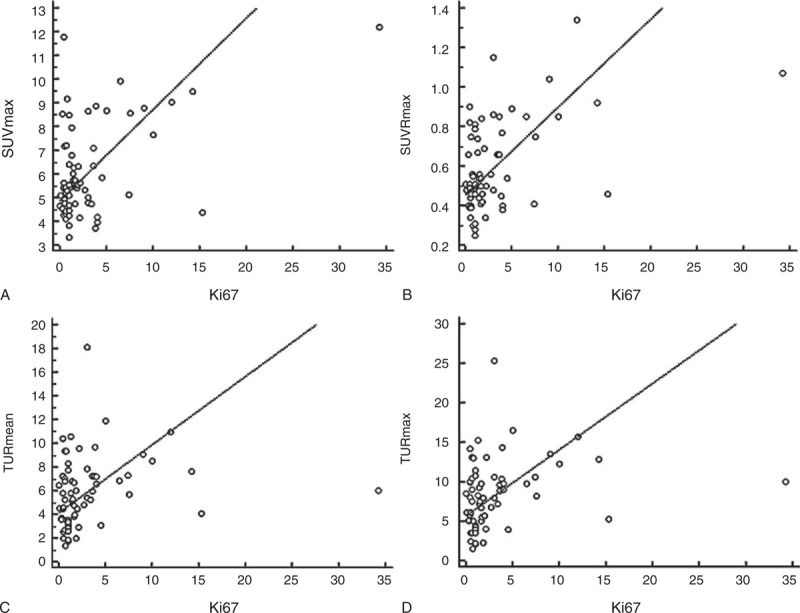
Scatter plots showing linear correlations of Ki-67 with (A) SUVmax, (B) SUVRmax, (C) TURmean, and (D) TURmax. All the correlations were statistically significant, but their coefficients were relatively low (r values were 0.273, 0.355, 0.322, and 0.336, respectively). SUV = standardized uptake value, SUVmax = the maximum value of SUV, SUVRmax = the maximum SUV ratio, TURmax = the maximum Tl uptake ratio, TURmean = the mean Tl uptake ratio.

Correlation to Ki-67 was statistically significant in all 4 indexes, but the coefficients were 0.273, 0.355, 0.322, and 0.336, for SUVmax, SUVRmax, TURmean, and TURmax, respectively, which were relatively low (Figure 4). No significant difference was observed among the coefficients.

## DISCUSSION

In this study, capability of Tl-SPECT and FDG-PET were compared for grading meningioma using 2 indexes for each method. In all indexes, significant difference was observed between low and high grades, and correlation to Ki-67 was statistically significant. AUC was the highest in TURmax, although no significant difference was observed among the indexes.

In FDG-PET, there was no significant difference between SUVmax and SUVRmax in AUCs and correlation to Ki-67. Both indexes can be used for meningioma grading, and SUV normalization by SUV of the normal gray matter was not considered mandatory. Previous studies have shown a correlation between FDG uptake of meningioma and its grades,^11–14^ which was the same in this study. Some studies reported that FDG uptake of meningioma showed no significant correlation with grades,^23–25^ but in these studies, enrolled patients were fewer than in this study. Furthermore, Cremerius et al demonstrated high sensitivity (89%) and specificity (88%) of FDG-PET in detecting high-grade meningioma.^11^ Lee et al showed that the cutoff value of 1.0 had sensitivity and specificity of 44% and 95%, respectively, when SUVmax to gray matter ratio was used.^14^ The result of our study using SUVmax is comparable to them with sensitivity 72.7% and specificity 87.5%.

Tl-SPECT imaging has also been used for grading meningioma.^15–18^ This scan can be conducted at early and late phases after Tl injection. For the early phase scan, difference in grading capability has been observed among previous studies. Kinuya et al^15^ reported positive capability, while others^16–18^ did not. In the latter studies, ROI selection was subjective and patient numbers enrolled were smaller than our study. Objective selection of ROIs using a semiautomatic threshold algorithm is also considered to contribute to the better results. Tl-SPECT scans at the early phase with objective analysis had high grading capability comparable to FDG-PET. Mean and maximum Tl uptake ratios were examined in this study. Mean uptake ratios have been frequently used,^15–18^ but this study found that maximum uptake ratios had slightly larger AUCs and higher correlation to Ki-67, although the difference was not statistically significant.

Thallium-201 is a potassium analogue possessing an affinity to the sodium- and potassium-activated adenosine triphosphatase (Na+-K+ ATPase) pump and is distributed in potassium-rich organs, such as heart, kidney, gastrointestinal tract, and thyroid gland, but Tl shows little uptake in the normal brain.^26^ Tl uptake in brain tumors depends on regional blood flow, permeability of the blood–brain barrier, and Na+-K+ ATPase activity in tumor cells.^27^ Tl uptake in low-grade meningioma was considered more related to lesion vascularity than Na+-K+ ATPase activity, whereas high-grade glioma had higher Na+-K+ ATPase activity than low-grade glioma.^28^ Both mechanisms may contribute to higher Tl uptake in high-grade meningioma than low-grade one.

In both AUCs and correlation to Ki-67, no statistically significant difference was observed among the 4 indexes. Even in the advanced countries, not many hospitals have access to an FDG-PET examination. Compared to PET scanners, much more SPECT scanners are distributed all over the world. This comparative study of Tl-SPECT and FDG-PET has shown that Tl-SPECT examination can be a sufficient alternative in surgical planning and guiding therapy for patients with intracranial meningioma.

Correlation of SUVRmax and Ki-67 in this study was almost the same to a previous result.^14^ Ki-67 was also significantly correlated with TURmax to the similar degree. A previous study found higher correlation between Tl uptake of delayed scans and Ki-67.^16^ In our study, delayed scans were not conducted, and if improvement in differential capability can be attained by using them or not cannot be investigated. This is the first limitation of this study. The other limitation is that recurrent cases were included, but this condition was the same for FDG-PET and Tl-SPECT examinations.

In conclusion, we confirmed that both FDG-PET and Tl-SPECT have high grading capability that was comparable to each other. Even if an FDG-PET examination is not available, a Tl-SPECT examination can take an equivalent role, and we may keep using it for grading meningioma.
